# Supplementary material for: Validation of the COmprehensive Score for Financial Toxicity (COST) in Vietnamese patients with cancer
Source: PLoS One. 2024 Jun 28;19(6):e0306339. doi: 10.1371/journal.pone.0306339 (PMC11213330; doi:10.1371/journal.pone.0306339)
Supplement: S1 File — (DOCX) [file pone.0306339.s001.docx]

**S1 Vietnamese version of COST**

| **Đánh giá sự phù hợp của các nhận định sau với tình trạng của ông/bà trong 7 ngày gần đây nhất** | | | | | | |
| --- | --- | --- | --- | --- | --- | --- |
|  | | **Hoàn toàn không đúng** | **Không đúng** | **Đúng một chút** | **Đúng** | **Rất đúng** |
| FT1 | Tôi biết rằng tôi có đủ tiền tiết kiệm, tiền hưu trí hoặc tài sản để trang trải chi phí điều trị của mình | 0 | 1 | 2 | 3 | 4 |
| FT2 | Chi phí y tế tự trả để điều trị bệnh của tôi nhiều hơn tôi nghĩ | 0 | 1 | 2 | 3 | 4 |
| FT3 | Tôi lo lắng về các vấn đề tài chính mà tôi sẽ gặp phải trong tương lai do bệnh tật hoặc quá trình điều trị bệnh của tôi | 0 | 1 | 2 | 3 | 4 |
| FT4 | Tôi cảm thấy tôi không có sự lựa chọn về chi phí dành cho quá trình điều trị bệnh | 0 | 1 | 2 | 3 | 4 |
| FT5 | Tôi cảm thấy thất vọng vì tôi không thể làm việc như trước đây | 0 | 1 | 2 | 3 | 4 |
| FT6 | Tôi hài lòng với tình hình tài chính hiện tại của mình | 0 | 1 | 2 | 3 | 4 |
| FT7 | Tôi có thể tự trang trải chi phí hàng tháng của mình | 0 | 1 | 2 | 3 | 4 |
| FT8 | Tôi đang cảm thấy căng thẳng về tài chính | 0 | 1 | 2 | 3 | 4 |
| FT9 | Tôi cảm thấy lo lắng về việc duy trì công việc và thu nhập của mình, bao gồm cả công việc ở nhà | 0 | 1 | 2 | 3 | 4 |
| FT10 | Bệnh ung thư hoặc việc điều trị của tôi đã làm giảm mức độ hài lòng của tôi với tình hình tài chính hiện tại của tôi | 0 | 1 | 2 | 3 | 4 |
| FT11 | Tôi cảm thấy kiểm soát được tình hình tài chính của mình | 0 | 1 | 2 | 3 | 4 |

Below is a list of statements that other people with your illness have said are important. **Please circle or mark one number per line to indicate your response as it applies to the past 7 days.**

|  | | **Not at all** | **A little bit** | **Somewhat** | **Quite a bit** | **Very much** |
| --- | --- | --- | --- | --- | --- | --- |
| FT1 | I know that I have enough money in savings, retirement, or assets to cover the costs of my treatment | 0 | 1 | 2 | 3 | 4 |
| FT2 | My out-of-pocket medical expenses are more than I thought they would be | 0 | 1 | 2 | 3 | 4 |
| FT3 | I worry about the financial problems I will have in the future as a result of my illness or treatment | 0 | 1 | 2 | 3 | 4 |
| FT4 | I feel I have no choice about the amount of money I spend on care | 0 | 1 | 2 | 3 | 4 |
| FT5 | I am frustrated that I cannot work or contribute as much as I usually do | 0 | 1 | 2 | 3 | 4 |
| FT6 | I am satisfied with my current financial situation | 0 | 1 | 2 | 3 | 4 |
| FT7 | I am able to meet my monthly expenses | 0 | 1 | 2 | 3 | 4 |
| FT8 | I feel financially stressed | 0 | 1 | 2 | 3 | 4 |
| FT9 | I am concerned about keeping my job and income, including paid work at home | 0 | 1 | 2 | 3 | 4 |
| FT10 | My cancer or treatment has reduced my satisfaction with my present financial situation | 0 | 1 | 2 | 3 | 4 |
| FT11 | I feel in control of my financial situation | 0 | 1 | 2 | 3 | 4 |
