# Supplementary material for: Validation of the COmprehensive Score for Financial Toxicity (COST) in Vietnamese patients with cancer
Source: PLoS One. 2024 Jun 28;19(6):e0306339. doi: 10.1371/journal.pone.0306339 (PMC11213330; doi:10.1371/journal.pone.0306339)
Supplement: Attachment — Submitted filename: 2_1 response to reviewers.docx [file pone.0306339.s004.docx]

Date: June 7^th^, 2024

Dear Editor and Reviewers

Thank you for your additional comments on our first-round revision. We have incorporated your feedback by merging it with the changes we made in the first round of revisions.

**Response to comments on May 27^th^, 2024 – Round 2**

Additional Editor Comments:

2.1. Please provide a detailed explanation of the revisions made to the document based on the opinions of two reviewers, using line by line format.

**Reply 2.1:** Thank you for your suggestion. We have added in the second part of this response letter and added page and line format

2. The reason for utilizing both Stata and Amos in the analysis of exploratory factor analysis (EFA) and confirmatory factor analysis (CFA) is to leverage the unique strengths and capabilities of each software tool. I am confident that STATA has the capability to perform these tasks.

**Reply 2.2:** Thank you again for your continued interest in our data analysis. While Stata was initially used for descriptive and multivariate analyses, we have reanalyzed the data using SPSS for consistency, as AMOS is also an SPSS extension. This information has been added on page 7, lines 130-131.

3. Do you believe that the presence of a patient aged 90-95 with an unusual value can significantly affect your results?
**Reply 2.3:** We have reviewed the data on age and found that the group of patients aged 80-95 represents only 1.67% of the total sample (result below). Therefore, we believe this distribution is unlikely to significantly influence our overall data analysis.

Age	Freq.	Percent	Cum.

27	1	0.33	0.33

32	2	0.67	1.00

33	1	0.33	1.33

34	2	0.67	2.00

35	2	0.67	2.67

36	1	0.33	3.00

38	5	1.67	4.67

39	5	1.67	6.33

40	7	2.33	8.67

41	1	0.33	9.00

42	6	2.00	11.00

43	3	1.00	12.00

44	2	0.67	12.67

45	3	1.00	13.67

46	4	1.33	15.00

47	6	2.00	17.00

48	4	1.33	18.33

49	7	2.33	20.67

50	9	3.00	23.67

51	5	1.67	25.33

52	6	2.00	27.33

53	8	2.67	30.00

54	9	3.00	33.00

55	11	3.67	36.67

56	10	3.33	40.00

57	13	4.33	44.33

58	12	4.00	48.33

59	7	2.33	50.67

60	8	2.67	53.33

61	10	3.33	56.67

62	12	4.00	60.67

63	10	3.33	64.00

64	12	4.00	68.00

65	12	4.00	72.00

66	13	4.33	76.33

67	15	5.00	81.33

68	7	2.33	83.67

69	6	2.00	85.67

70	8	2.67	88.33

71	5	1.67	90.00

72	8	2.67	92.67

73	5	1.67	94.33

74	1	0.33	94.67

75	3	1.00	95.67

77	1	0.33	96.00

78	1	0.33	96.33

79	3	1.00	97.33

80	3	1.00	98.33

83	2	0.67	99.00

86	2	0.67	99.67

95	1	0.33	100.00

Total	300	100.00

.

4. Please find some english certificate group that can assist you in preparing good english manuscript

**Reply 2.4.** Thank you for your comments on the language of our manuscript. We have addressed them by utilizing a professional English editing service (Editage) during this second round of revisions. Please find the certificate attached.

**Response to comments on May 15^th^, 2024 (Editors, reviewer 1 & reviewer 2) – Round 1**

**The title of study
All:** We have modified the title of study as “Validation of the COmprehensive Score for Financial Toxicity (COST) in Vietnamese Patients with Cancer”**. (**page 1, line 1-2*)*

**Reviewer 1**

Reviewer #1: The paper offers a comprehensive validation study of the Vietnamese version of the Comprehensive Score for Financial Toxicity (COST) instrument among cancer patients. While the methodology is well-described and the study design seems robust, there are formatting inconsistencies and minor issues that require attention before publication.

1. Throughout the manuscript, there are multiple variations of the term for the Vietnamese version of COST. It is crucial to use only one consistent term for clarity. Please ensure that the same term is used consistently across the entire manuscript.

**Reply 1:** We have corrected all the terms used for COST, Financial toxicity and others statistical analysis.

2. The paragraph format in the statistical analysis section lacks consistency. It is suggested to remove the headings "Descriptive statistics" and "Internal consistency" to maintain coherence.

**Reply 2:** We have modified this part for consistency. (page 5-6, line 106-129)

Statistical analysis

Continuous variables were summarized using means, standard deviations (SD), median, and Interquartile range (IQR). Categorical variables were described using frequencies and percentages.

The reliability of the scale was assessed using Cronbach's alpha and McDonald's omega coefficients. A coefficient of 0.7 or higher generally indicates good internal consistency [24].

Construct validity involved two steps including exploratory factor analysis (EFA) and confirmatory structure analysis (CFA). EFA identified underlying factors influencing questionnaire responses. The scree plot and varimax rotation determined the number of factors retained [25]. EFA ensured clear and reliable factors through several strategies: (1) Removing items with low loadings (< 0.4) for minimal contribution; (2) requiring factors to have at least 3 items to capture the underlying construct adequately; (3) Removing items with high cross-loadings (> 0.3) to minimize ambiguity and enhance factor distinction [26]. Bartlett's sphericity test and Kaiser-Meyer-Olkin (KMO) confirmed the suitability of EFA [27, 28]. Building on the initial exploration of the factor structure using EFA (two factors loading was selected), CFA was then employed to assess the validity of the identified factor structure [27, 29]. Several fit indices (Root Mean Square Error of Approximation (RMSEA), Goodness-of-Fit Index (GFI), Comparative Fit Index (CFI), and Adjusted Goodness-of-Fit Index (AGFI)) indicated a good model fit (p < 0.001).

Convergent validity was assessed using the Pearson correlation coefficient between the COST score and HRQoL (EQ-5D and EQ VAS). Shapiro-Wilk W test (p-value < 0.001) indicated that the COST score were not normally distributed due to skewness. Therefore, nonparametric tests were used to analyze the differences in COST score by characteristics. The Mann-Whitney U test was used for comparisons between two groups, and the Kruskal-Wallis test was used for comparisons between three or more groups. Additionally, a multivariate quantile regression analysis (at the 50th percentile) was conducted to investigate factors associated with the COST score. All independent variables were included for adjustment in the model. The threshold of a statistically significant level was less than 0.05.

3. Recheck the format of references and citations to ensure consistency and accuracy. Pay attention to punctuation, capitalization, and formatting style (e.g., APA, MLA) to adhere to journal guidelines.

**Reply 3:** We have checked and corrected all citations and references in compliance with journal requirement.

4. The term "Exploratory factor analysis" is redundant as it has already been explained previously. Consider removing it for clarity and conciseness.

**Reply 4:** Thank you for your thorough review. We have removed duplicate information from the statistical analysis and revised the entire section accordingly. (see reply 2, page 6, line 110-129)

5. Additional Comments: Please double-check for any formatting errors or inconsistencies throughout the manuscript to maintain readability.

**Reply 5:** Thank you again for your suggestion. We have carefully reviewed the document and made substantial revisions.

**Reviewer 2**

Reviewer #2: The study “Validation of the Vietnamese Comprehensive Score for Financial Toxicity (COST) in Cancer Patients” was performed by Tran et al.
The paper is well-written and showed that the COmprehensive Score for financial Toxicity (COST) demonstrated good internal consistency and The Vietnamese COST is reliable and valid.
There were several unclear issues and limitations that need to be addressed:

6. For the principal component analysis, two factors explained more than 64% of the variance, that a low explanation. With this analysis, you can consider adding more factors to have more than 80% of the variance.

**Reply 6:** Thank you for raising a critical point regarding the validation steps. After carefully checking our data and re-analyzing it, we found that achieving over 80% variance requires at least five factors to load. (Page 12, line 162-168)

Unfortunately, this is not ideal for our scale with only 11 items. We have addressed this limitation in the discussion section on page 10 to further explain our results.

However, in our data analysis, achieving an explained variance of over 80% required five-factor loadings, which is not ideal for the COST scale with only 11 items. Our final two-factor model demonstrated acceptable fit indices consistent with the findings of Yu et al. in China [15]. These comparisons suggest that the COST is likely to measure distinct but related constructs relevant to FT in patients with cancer. (Page 18, line 233-237)

We have copied output from AMOS for reference

| **Total Variance Explained** | | | | | | | | | | |
| --- | --- | --- | --- | --- | --- | --- | --- | --- | --- | --- |
| Component | Initial Eigenvalues | | | Extraction Sums of Squared Loadings | | | | Rotation Sums of Squared Loadings | | |
|  | Total | % of Variance | Cumulative % | | Total | % of Variance | Cumulative % | Total | % of Variance | Cumulative % |
| 1 | 6.003 | 54.572 | 54.572 | | 6.003 | 54.572 | 54.572 | 3.747 | 34.067 | 34.067 |
| 2 | 1.135 | 10.317 | 64.889 | | 1.135 | 10.317 | 64.889 | 3.390 | 30.822 | 64.889 |
| 3 | .724 | 6.582 | 71.471 | |  |  |  |  |  |  |
| 4 | .645 | 5.863 | 77.334 | |  |  |  |  |  |  |
| 5 | .566 | 5.145 | 82.479 | |  |  |  |  |  |  |
| 6 | .486 | 4.415 | 86.894 | |  |  |  |  |  |  |
| 7 | .382 | 3.472 | 90.366 | |  |  |  |  |  |  |
| 8 | .346 | 3.148 | 93.514 | |  |  |  |  |  |  |
| 9 | .297 | 2.704 | 96.218 | |  |  |  |  |  |  |
| 10 | .269 | 2.443 | 98.661 | |  |  |  |  |  |  |
| 11 | .147 | 1.339 | 100.000 | |  |  |  |  |  |  |
| Extraction Method: Principal Component Analysis. | | | | | | | | | | |

7. Why did the authors express the COST score by both Mean ± SD and Median? This should be based on the distribution of COST score in each group, normal or non-normal distribution. Please also verify the statistical analysis, which tests were used to analyze the differences of COST score: t-test, ANOVA, Kruskal-Wallis or Mann Whitney?

**Reply 7:** Thank you for identifying the issues in our statistical analysis. We have addressed these concerns by revising the statistical method and incorporating the median, IQR (interquartile range), and p-value into Table 1.

Statistical method (Page 6, line 122-129):

Convergent validity was assessed using the Pearson correlation coefficient between the COST score and HRQoL (EQ-5D and EQ VAS). Shapiro-Wilk W test (p-value < 0.001) indicated that the COST scores were not normally distributed due to skewness. Therefore, nonparametric tests were used to analyze the differences in COST score by characteristics. The Mann-Whitney U test was used for comparisons between two groups, and the Kruskal-Wallis test was used for comparisons between three or more groups. Additionally, a multivariate quantile regression analysis (at the 50th percentile) was conducted to investigate factors associated with the COST score. All independent variables were included for adjustment in the model. The threshold of a statistically significant level was less than 0.05.

Table 1:

| **Chracteristics** | **N (%)** | **COST score Mean±SD** | **Median** | **IQR (25^th^ -75^th^ )** | ***p* value*** |
| --- | --- | --- | --- | --- | --- |
| All | 300 | 15.1 (7.3) | 13 | 11-18 |  |
| Age |  |  |  |  | 0.0747 |
| < 60 | 152 (50.7) | 14.1 (6.2) | 13 | 11-16.5 |  |
| ≥ 60 | 148 (49.3) | 16.2 (8.2) | 14 | 11-20.5 |  |
| Mean±SD  (Max – Min) | 58.5±11.9 |  |  |  |  |
| Sex |  |  |  |  | 0.0121 |
| Men | 208 (69.3) | 15.8 (7.8) | 14 | 11-20 |  |
| Women | 92 (30.7) | 13.4 (6.0) | 13 | 9.5-17 |  |
| Education level |  |  |  |  | 0.0030 |
| Less than secondary school | 95 (31.7) | 13.5 (6.1) | 12 | 11-15 |  |
| From secondary school and above | 205 (68.3) | 15.8 (7.8) | 14 | 11-20 |  |
| Living areas |  |  |  |  | 0.0031 |
| Urban | 226 (75.3) | 14.3 (6.8) | 13 | 11-17 |  |
| Rural | 74 (24.7) | 17.7 (8.3) | 15 | 12-23 |  |
| Religious |  |  |  |  | 0.288 |
| No | 248 (82.7) | 15.2 (7.5) | 13 | 11-18 |  |
| Yes | 52 (17.3) | 14.4 (6.4) | 13 | 11-17.5 |  |
| Marital status |  |  |  |  | 0.961 |
| Married | 277 (92.3) | 15.1 (7.4) | 13 | 11-18 |  |
| Single/Widow/Dervoce | 23 (7.7) | 14.7 (7.1) | 14 | 9-19 |  |
| Occupation |  |  |  |  | 0.0001 |
| Stable income jobs | 66 (22.0) | 18.8 (9.2) | 16.5 | 13-23 |  |
| Unstable income jobs | 163 (54.3) | 13.5 (6.5) | 13 | 10-16 |  |
| No income | 71 (23.7) | 15.4 (6) | 14 | 11-19 |  |
| Economic status |  |  |  |  | 0.0070 |
| Poor | 19 (6.3) | 11.5 (6.0) | 11 | 8-13 |  |
| Near poor | 24 (8.0) | 13.1 (6.2) | 12 | 11-15.5 |  |
| Average | 257 (85.7) | 15.6 (7.4) | 13 | 11-19 |  |
| Health insurance benefits |  |  |  |  | 0.5507 |
| 100% | 153 (51.0) | 15.4 (8.1) | 13 | 11-19 |  |
| 95% | 36 (12.0) | 15.9 (6.6) | 14 | 11.5-20.5 |  |
| 80% | 111 (37.0) | 14.4 (6.5) | 13 | 11-17 |  |
| Type of cancers |  |  |  |  | 0.0252 |
| Oesophagus cancer | 39 (13.0) | 12.3 (6.0) | 12 | 8-15 |  |
| Stomach cancer | 15 (5.0) | 13.3 (3.3) | 13 | 13-16 |  |
| Colorectal cancer | 30 (10.0) | 15.9 (7.2) | 13 | 11-22 |  |
| Liver cancer | 32 (10.7) | 15.4 (7.1) | 15 | 10-18.5 |  |
| Biliary/pancreatic cancer | 112 (37.3) | 16.6 (8.3) | 14 | 11-21 |  |
| Breast cancer | 72 (24.0) | 14.2 (6.8) | 13 | 11-15 |  |
| Stages of cancer |  |  |  |  | 0.343 |
| I | 6 (2.0) | 12.3 (2.8) | 11.5 | 11-14 |  |
| II | 54 (18.0) | 13.8 (7.8) | 13 | 9-18 |  |
| III | 110 (36.7) | 15.2 (7.1) | 13 | 11-16 |  |
| IV | 130 (43.3) | 15.7 (7.4) | 13 | 11-20 |  |
| Treatment modality |  |  |  |  | 0.0385 |
| Surgery | 4 (1.3) | 18.5 (17.4) | 12.5 | 8.5-28.5 |  |
| Surgery and chemotherapy | 153 (51.0) | 16.3 (8.0) | 14 | 11-20 |  |
| Chemotherapy | 83 (27.7) | 13.2 (5.8) | 13 | 10-16 |  |
| Radiotherapy | 54 (18.0) | 15 (6.3) | 13 | 11-17 |  |
| Palliative care | 6 (2.0) | 10.5 (4.0) | 10.5 | 8-14 |  |
| Alcohol use |  |  |  |  | 0.670 |
| Yes | 175 (58.3) | 14.9 (7.3) | 13 | 11-17 |  |
| No | 125 (41.7) | 15.4 (7.4) | 13 | 11-19 |  |
| Cigarette status |  |  |  | 11-17 | 0.953 |
| Yes | 146 (48.7) | 14.9 (6.8) | 13 | 10-19 |  |
| No | 154 (51.3) | 15.3 (7.8) | 13 | 11-17 |  |

8. A linear regression should be performed to understand the contribution of independent variables to the COST score.

**Reply 8:** We appreciate your suggestion once again. To address the skewed distribution of the COST score, we performed a multivariate quantile regression model to identify its predictors. We focused on the 50th percentile (median) of the COST score in this regression analysis. All results are presented in Table 4 and the results section (Page 15, line 196-197).

Table 4. A multivariate quantile regression analysis for predictors of COST score.

| **Factors** | **Coefficient** | **[95% CI]** | **p-value** |
| --- | --- | --- | --- |
| Age group (≥ 60 vs. <60) | 2.50 | -0.12 to 5.12 | 0.062 |
| Sex (*Women vs. Men*) | -5.96 | -10.39 to -1.54 | 0.008 |
| Education level (*From secondary school and above vs.Less than secondary school*) | 0.39 | -2.46 to 3.24 | 0.786 |
| Residence areas *(Rural vs. Urban )* | -4.57 | -7.41 to 1.73 | 0.002 |
| Religious *(Yes vs. No)* | -1.50 | -4.74 to 1.74 | 0.363 |
| Marital status (*Yes vs. No*) | 1.11 | -3.44 to 5.65 | 0.632 |
| Occupation (*vs. Stable income jobs*) |  |  |  |
| Unstable income jobs | -5.39 | -8.71 to -2.07 | 0.002 |
| No income | -4.64 | -8.63 to -0.65 | 0.023 |
| Family economic status *(vs. Poor)* |  |  |  |
| Near poor | 0.82 | -5.58 to 7.23 | 0.801 |
| Average | 2.96 | -2.1 to 8.03 | 0.250 |
| Health insurance copayment (*vs. 100%)* |  |  |  |
| 95% | -4.07 | -8.35 to 0.2 | 0.062 |
| 80% | -0.86 | -3.49 to 1.78 | 0.522 |
| Types of cancer (*vs. Oesophagus cancer*) |  |  |  |
| Stomach cancer | 1.29 | -6.64 to 9.21 | 0.750 |
| Colorectal cancer | 2.68 | -3.32 to 8.68 | 0.380 |
| Liver cancer | 3.00 | -2.39 to 8.39 | 0.274 |
| Biliary/pancreatic cancer | 2.04 | -2.77 to 6.84 | 0.405 |
| Breast cancer | 0.07 | -5.66 to 5.8 | 0.980 |
| Stages of cancer *(vs. Stage I)* |  |  |  |
| II | 4.18 | -4.63 to 12.99 | 0.351 |
| III | 4.36 | -4.19 to 12.91 | 0.317 |
| IV | 5.11 | -3.52 to 13.74 | 0.245 |
| Treatment modality (*vs. Surgery*) |  |  |  |
| Surgery and chemotherapy | 1.68 | -8.87 to 12.23 | 0.754 |
| Chemotherapy | -0.25 | -10.8 to 10.3 | 0.963 |
| Radiotherapy | 0.11 | -10.85 to 11.06 | 0.985 |
| Palliative care | -5.54 | -18.94 to 7.87 | 0.417 |
| Alcohol use (*No vs. Yes*) | 2.86 | -0.64 to 6.36 | 0.109 |
| Cigarette smoking status (*No vs. Yes*) | 2.36 | -0.98 to 5.69 | 0.165 |

CI, Confidence Interval
